# Enhancing resilient coping strategies for quality of life in Chinese adult children caregiving for parents with advanced cancer: a cross-sectional study

**DOI:** 10.1007/s00520-023-08057-y

**Published:** 2023-09-26

**Authors:** Xian Chen, Chengping Qiao, Anne Arber, Yan Shen, Yehao Rui, Rui Zhang, Zhongling Pei, Yuanyuan Tang, Ziyu Sha, Jianying Bao, Jia Zhang, Jinhua Li, Dan Wang, Xuemei Wang

**Affiliations:** 1 Gynaecology Department, Women’s Hospital of Nanjing Medical University (Nanjing Maternity and Child Health Care Hospital), Nanjing, People’s Republic of China; 2 School of Health Sciences, Faculty of Health and Medical Sciences, The University of Surrey, Guildford, UK; 3 Interventional Radiology Department, The First Affiliated Hospital With Nanjing Medical University, Nanjing, People’s Republic of China; 4 Oncology Department, Zhongda Hospital Southeast University, Nanjing, People’s Republic of China; 5 Oncology Department, The Affiliated Hospital of Xuzhou Medical University, Xuzhou, People’s Republic of China; 6 Radiotherapy Department, The First Affiliated Hospital With Nanjing Medical University, Nanjing, People’s Republic of China; 7 Oncology Department, The First Affiliated Hospital With Nanjing Medical University, Nanjing, People’s Republic of China; 8 Geriatric Oncology Department, The First Affiliated Hospital With Nanjing Medical University, Nanjing, People’s Republic of China

**Keywords:** Caregiver burden, Quality of life, Individual resilience, Anxiety, Depression, Adult child, Parental advanced cancer

## Abstract

**Purpose:**

This study investigated the mediating role of individual resilience in the relationship between caregiver burden and quality of life (QoL) among Chinese adult children providing care to their parents with advanced cancer, with the aim to inform effective coping strategies and positive caregiving outcomes.

**Methods:**

In a cross-sectional design, 614 caregivers from multiple centers, whose parents were undergoing chemotherapy and/or radiotherapy, completed questionnaires encompassing demographics, caregiver burden, symptoms of anxiety and depression, resilience, and QoL.

**Results:**

Findings revealed a moderate level of caregiver burden among participants, significantly influenced by factors including education level, family income, single-child status, and participation in social media patient support groups. Caregivers who were only children or involved in patient support groups reported higher burden. Importantly, path analysis showed a significant impact of caregiver burden, anxiety, and depression on QoL, with these relationships being mediated by individual resilience.

**Conclusions:**

Chinese adult child caregivers face a considerable burden, negatively influencing their QoL. Individual resilience, a modifiable factor, was identified as a critical mediator in this relationship, mitigating the negative implications of caregiver burden, anxiety, and depression. These findings underscore the need for caregiver interventions that consider not only demographics but also the socio-psychological dynamics of caregiving to enhance caregiver QoL.

**Supplementary Information:**

The online version contains supplementary material available at 10.1007/s00520-023-08057-y.

## Introduction

Cancer is one of the leading public health issues worldwide. Recent advancements in cancer treatment and supportive care have led to a growing number of cancer survivors and their families coping with the disease and its effects over extended periods [1]. The impact of cancer on the family members of survivors is an integral part of cancer survivorship, as cancer is seldom experienced in isolation [2]. While research has predominantly focused on the adverse health effects and emotional burden of caregiving on spouses and young children (including adolescents), who may be less equipped to handle the demands of caregiving, it is important to note that most cancer cases occur in individuals over the age of sixty whose children are likely to be adults [3]. Consequently, adult children of all ages are frequently called upon to care for their parents with cancer, whether willingly or not. The effects of providing care for parents can be burdensome for adult children.

Caregiver burden refers to the emotional, social, and financial strain experienced by caregivers as a result of their caregiving responsibilities [4]. Numerous studies have demonstrated that family caregivers, including adult children, experience a significant burden when providing care to patients with chronic diseases, such as cancer [5–7]. Adult children who are caregivers of parents with cancer face numerous challenges, including fear of loss, emotional isolation, supporting the patient, making treatment decisions, and managing care-related stress and future uncertainties [3, 8, 9]. Additionally, parental cancer can raise concerns about the children's genetic risk of the disease and mortality. Caregiver burden is particularly high in advanced cancer, as the disease is unlikely to be cured or controlled with treatment [10, 11]. The burden of caring can negatively impact the psychological and physical well-being of the adult child caregiver [5, 12].

Caregiver burden can lead to feelings of anxiety and depression, which are common mental health conditions among caregivers [7]. Anxiety is characterized by feelings of fear, worry, and nervousness, while depression is characterized by feelings of sadness, hopelessness, and a loss of interest in activities that were once enjoyable. Both anxiety and depression can be triggered by the stress and demands of caregiving [13]. Caregivers may find it difficult to cope with their responsibilities and feel overwhelmed and exhausted. Therefore, they may feel anxious and depressed which can have a negative impact on the caregiver's quality of life (QoL). For instance, according to Song et al. [11], family caregivers of patients with terminal cancer experience mental health problems and deterioration of health-related QoL.

Psychological disorders are not the sole outcomes of caregiver burden in adult child caregivers. Some caregivers may experience positive adaptations, leading to better QoL despite the challenges [14, 15]. This phenomenon, known as individual resilience, involves effectively coping with adversity and achieving positive outcomes. Developing resilience is a dynamic process that utilizes psychological resources like positive thinking, problem-solving, social support, and emotional regulation. It also involves employing adaptive coping mechanisms and stress management techniques. Research suggests that some adult child caregivers develop individual resilience, such as positive emotions and seeking support, in response to caregiving challenges, creating a more protective environment [16, 17]. However, there is substantial variation in caregiver outcomes influenced by various factors, including gender and personality attributes like optimism, self-esteem, and self-mastery [17, 18]. These attributes can enhance resilience by fostering a positive mindset, boosting self-confidence, and improving problem-solving skills. Adult children with higher resilience levels may have better QoL. Despite this, the mediating effects of individual resilience on caregiver burden outcomes in adult children caring for parents with cancer are understudied.

China, as one of the most populous countries in the world, has experienced a substantial increase in the prevalence and burden of cancer [19].The age-standardized cancer incidence rate in China showed a general rising trend from 2000 to 2011, with a notable increase among females and a stable pattern among males [20]. In particular, the age-standardized incidence rates of colorectal cancer exhibited a significant upward trend for both genders. For males, the age-standardized incidence rate of prostate cancer also rose markedly, with an annual percentage change of 12.6% from 2000 to 2011. The age-standardized incidence rates of breast, cervical, and ovarian cancers increased sharply as well [20]. Adult child caregivers in China face considerable psychological pressure due to the traditional notion of raising children for old-age support. The situation is further complicated by the prevalence of single-child families, a result of the four-decade-long family planning policy since the 1970s [21]. In light of these factors, our study aimed to examine the mediating effects of individual resilience on the relationship between caregiver burden and QoL among Chinese adult children caring for parents with advanced cancer. Firstly, we hypothesized that adult child caregivers of parents with advanced cancer in China experience a certain level of caregiver burden. Secondly, we hypothesized that higher caregiver burden is associated with poorer QoL. Lastly, we hypothesized that individual resilience serves as a modifiable positive factor that mediates the relationship between caregiver burden and QoL, counterbalancing the negative impact of factors like anxiety and depression.

## Method

### Study design and participants

This cross-sectional, multicenter study was conducted at four affiliated hospitals of universities in Jiangsu Province China from March to November 2021. A convenience sample of relative caregivers of cancer patients was included in this study. All patients were diagnosed with advanced cancer and were admitted to the hospital for chemotherapy and/or radiotherapy treatment in the oncology department.

Ethical approval was obtained from the First Affiliated Hospital of Nanjing Medical University Institutional Review Board (2020-SR-253). Informed consent was obtained from all study participants, who were made aware of their right to withdraw from the study for any reasons at any time. All data collected during the study were kept confidential and anonymous, with access limited to the research team. Steps were taken to ensure the privacy and confidentiality of the participants’ data, and any identifying information was removed before analysis.

**Fig. 1:**
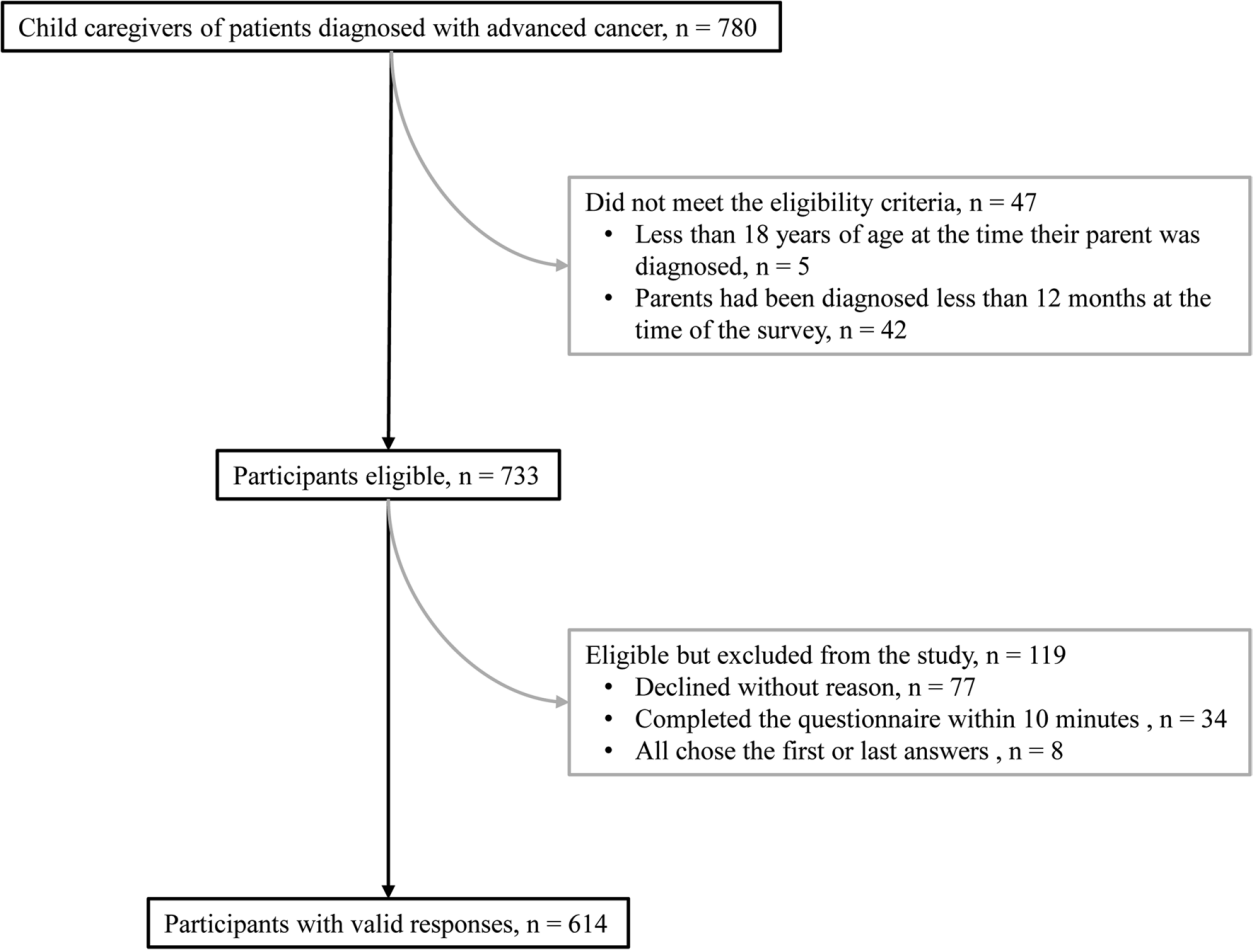
Flowchart of participants

The inclusion criteria for caregivers in this study were as follows: (1) they were 18 years of age or older at the time their parent was diagnosed with cancer; (2) their parent had been diagnosed with cancer at least 12 months prior to their participation in the study; and (3) their parent had advanced cancer (presence of distant metastases or regional recurrence) at the time of conducting the survey. Reasons for not taking part in the study included completing the questionnaire too quick (within 10 min), all answers were the first or last options, and declining to participate for no reason (see Fig. 1 for the flowchart of participants).

### Measures

#### Demographics

Demographic questionnaire asked participants for information on age, gender, education level, marital status, monthly income, presence of any chronic diseases, caring duration, whether they are an only child, and whether they have participated in online health support groups.

#### Hospital anxiety and depression scale (HADS)

The HADS was utilized in this study to evaluate symptoms of anxiety and depression [22]. The HADS is a self-reported instrument with 14 items that assess anxiety and depressive symptoms in populations with cancer caregivers [23, 24]. It consists of two subscales, each with seven items: HADS Depression (HD) and HADS Anxiety (HA). Each item is rated on a 4-point scale (0 = not at all to 3 = nearly all the time). The score range for each subscale is 0–21. Scores are interpreted as follows: no symptoms (0–7), mild symptoms (8–10), moderate symptoms (11–14), or high symptoms (15–21). A higher score indicates a higher level of anxiety and depression. The internal consistency of the HADS in this study was satisfactory, with a Cronbach's alpha of 0.77.

#### Chinese version of the Zarit caregiver burden interview (CZBI)

The CZBI was used in this study to evaluate the burden experienced by family caregivers who care for patients in the hospital [25]. The CZBI is composed of 22 items, with each item rated on a five-point scale ranging from 0 (never) to 4 (nearly always). The total scores range from 0 to 88, with higher scores indicating an increased caregiver burden. The degree of caregiver burden was divided into four categories: 0 to 20 (little or no burden), 21 to 40 (mild to moderate burden), 41 to 60 (moderate to severe burden), and 61 to 88 (severe burden). The CZBI is a validated and practical instrument, with a Cronbach's α of 0.92 in this study.

#### Chinese version of the Conner-Davidson resilience scale (CD-RISC)

The CD-RISC was used in this study as a self-report instrument to assess resilience among caregivers. The CD-RISC consists of 25 items, and each item is scored on a five-point Likert scale ranging from 0 (not at all) to 4 (true nearly all the time) [26]. The total score ranges from 0 to 100, with higher scores indicating greater resilience. The CD-RISC has been validated for situations of chronic stress [27] and has demonstrated good reliability in this study, with a Cronbach's α of 0.94.

#### Twelve-item short form health survey (SF-12)

The SF-12 was employed in this study to gather data on participants' QoL. The SF-12 is scored using a weighted algorithm, and scores range from 0 to 100 for each of the two summary scales: the Physical Component Summary (PCS) and the Mental Component Summary (MCS) [28]. Higher scores on these scales indicate better QoL, with a score of 50 representing the average for the general population. It has been shown to be a valid and reliable measure of QoL in various populations, including those with chronic diseases, cancer, and mental health disorders [29–31]. The Cronbach's alpha coefficient for the SF-12 in this study was 0.846, indicating good internal consistency.

### Procedure

Trained nurses explained the purpose of the study to all the participants. Only those who agreed to participate in the study were required to complete the written informed consent. The participants were asked to complete the pen-and-paper self-report questionnaire in a quiet room during the patients' hospitalization. It took approximately 30 min to complete the entire questionnaire.

### Statistical analyses

SPSS 22.0 software was used for data cleaning and analysis in this study. Descriptive statistics, including mean and standard deviation, were used to describe normally distributed metric data. Two independent sample t-tests and analysis of variance (ANOVA) were used for intergroup comparisons, and the SNK method was used for pairwise comparisons. Pearson correlation analysis was used to investigate the correlations between variables. Amos 24.0 software was used to construct a mediation model, and the maximum likelihood method was used to estimate the model parameters. The model was adjusted based on the Modification Indices. A good fit was considered to be achieved when the ratio of chi-square to degrees of freedom was less than 5.0, the root mean square error of approximation (RMSEA) was less than 0.08, and the goodness-of-fit index (GFI), incremental fit index (IFI), and confirmatory fit index (CFI) were all greater than or equal to 0.90. The bias-corrected percentile bootstrap method (with 5,000 repeated samples) was used to calculate the confidence intervals of the effects. The significance level was set at α = 0.05.

## Results

### Sample characteristics

The descriptive statistics for all study variables are shown in Table 1. The participants in this study were primarily caregivers for parents suffering from advanced cancer. The mean age of these caregivers was 49 years, ranging from 26 to 74 years. The mean score of caregiver burden of all participants was 28.25 ± 16.21, indicating moderate caregiver burden among adult children with advanced parental cancer.

**Table 1:** Descriptive statistics and differences in caregiver burden and quality of life by sociodemographic variables (*n* = 614)

Variables	Number (%)	Care burden	*t/F*	*P*	PCS-12	*t/F*	*P*	MCS-12	*t/F*	*P*
Age (years)			1.86	0.16		7.03	< 0.01		4.72	< 0.01
< 40	195 (31.76)	30.09 ± 17.36			47.21 ± 8.92			46.49 ± 9.05		
40 ~ 50	155 (25.24)	27.26 ± 15.33			47.81 ± 7.97			49.40 ± 8.61^a^		
> 50	264 (43)	27.46 ± 15.77			44.88 ± 8.74^ab^			48.16 ± 9.03		
Sex			1.09	0.28		-0.52	0.60		2.235	0.03
Male	273 (44.46)	29.04 ± 16.52			46.16 ± 8.82			48.84 ± 8.81		
Female	341 (55.54)	27.61 ± 15.95			46.52 ± 8.60			47.22 ± 9.08		
Education			8.98	< 0.01		1.29	0.28		2.15	0.12
Primary education	205 (33.39)	31.91 ± 17.91			45.56 ± 8.79			48.03 ± 8.37		
Secondary education	190 (30.94)	26.00 ± 15.95^c^			46.74 ± 7.92			48.88 ± 9.33		
Bachelor’s level and above	219 (35.67)	26.45 ± 13.53^c^			46.78 ± 9.22			47.04 ± 9.19		
Caring duration (years)			-0.14	0.89		1.23	0.22		1.60	0.11
≤ 2	428 (69.71)	28.18 ± 16.55			46.66 ± 8.32			48.32 ± 8.64		
> 2	186 (30.29)	28.39 ± 15.42			45.67 ± 9.49			47.06 ± 9.72		
Marital status			-0.64	0.53		0.76	0.45		2.47	0.01
Married	576 (93.81)	28.16 ± 16.44			46.43 ± 8.61			48.17 ± 9.01		
Others	38 (6.19)	29.50 ± 12.23			45.32 ± 10.02			44.47 ± 7.99		
Family income (RMB/Month)			3.31	0.02		0.54	0.66		1.92	0.13
≤ 2000	152 (24.75)	30.09 ± 17.71^d^			47.03 ± 8.21			48.21 ± 9.35		
2001—5000	229 (37.30)	28.87 ± 14.7^d^			46.4 ± 8.95			47.33 ± 9.12		
5001—8000	144 (23.45)	29.47 ± 15.89^d^			45.96 ± 8.71			49.29 ± 8.23		
> 8000	89 (14.50)	24.73 ± 16.76			45.76 ± 8.88			46.86 ± 9.06		
Only children			3.32	< 0.01		-0.84	0.40		-3.75	< 0.01
Yes	81 (13.19)	33.77 ± 17.96			45.61 ± 9.9			44.48 ± 9.53		
No	533 (86.81)	27.41 ± 15.77			46.47 ± 8.5			48.46 ± 8.80		
Chronic disease			0.72	0.47		-3.47	< 0.01		-0.601	0.548
Yes	202 (32.9)	28.88 ± 14.56			44.64 ± 8.65			47.63 ± 9.11		
No	412 (67.1)	27.93 ± 16.97			47.21 ± 8.60			48.09 ± 8.94		
Patient support group on social media			3.31	< 0.01		0.42	0.68		-3.466	< 0.01
Yes	101 (16.45)	33.08 ± 17.25			46.69 ± 8.2			45.13 ± 9.08		
No	513 (83.55)	27.29 ± 15.84			46.3 ± 8.8			48.49 ± 8.88		

*PCS-12* physical component summary, *MCS-12* mental component summary

^a^ compared with age < 40, *P* < 0.05; ^b^ compared with age 40 ~ 50 *P* < 0.05

^c^ compared with primary education, *P* < 0.05

^d^ compared with family income > 8000, *P* < 0.05

There were significant differences in caregiver burden based on education level, family income, only child status, and participation in a patient support group on social media (Table 1). Post hoc tests revealed that caregivers who were only children experienced a higher burden (33.77 ± 17.96) compared to those who were not the only child in the family (27.41 ± 15.77). The burden was also significantly higher among caregivers who were members of patient support groups on social media (33.08 ± 17.25) versus those who were not (27.29 ± 15.84). With respect to education, caregivers with a primary education level experienced a notably higher burden (31.91 ± 17.91) than those with secondary education (26.00 ± 15.95) or a bachelor’s degree and above (26.45 ± 13.53). Similarly, caregivers with a family income of 8000 RMB or more per month experienced a lower burden (24.73 ± 16.76) compared to those in other income brackets. However, there were no significant differences in caregiver burden according to age, sex, caring duration, marital status, or presence of a chronic disease.

The mean score of PCS-12 and MCS-12 among adult children was 46.36 ± 8.70 and 47.94 ± 8.99 respectively. The PCS-12 score was lower in participants with older age and in those with presence of chronic disease. The MCS-12 score, on the other hand, was significantly lower in participants who had the following characteristics: less than 40 years old, female, not married, only children, and participated in a virtual patient support group.

### Correlations among caregiver burden, hospital anxiety, hospital depression, individual resilience, and QoL (PCS-12 and MCS-12)

The Pearson’s correlation analysis (Table 2) revealed several notable relationships between the variables in this study. Both HA and HD showed a positive correlation with caregiver burden (r = 0.328 and 0.194, respectively, *p* < 0.001), and a negative correlation with resilience and PCS-12 scores (r = -0.348 to -0.261, *p* < 0.001). This indicates that higher levels of anxiety and depression were associated with higher caregiver burden and lower resilience and physical health-related QoL. Resilience, on the other hand, was positively correlated with PCS-12 scores (r = 0.190, *p* < 0.001), suggesting that higher resilience was associated with better physical health-related QoL.

**Table 2:** Correlations between HA, HD, Care burden, Resilience, PCS-12 and MCS-12 (*N* = 614)

	Mean ± SD	HA	HD	Care burden	Resilience	PCS-12
HA	8.76 ± 2.84	1				
HD	7.87 ± 2.86	0.430*	1			
Care burden	28.25 ± 16.21	0.328*	0.194*	1		
Resilience	66.56 ± 17.37	-0.348*	-0.363*	-0.027	1	
PCS-12	46.36 ± 8.7	-0.265*	-0.261*	-0.377*	0.190*	1
MCS-12	47.94 ± 8.99	-0.520*	-0.370*	-0.377*	0.314*	0.290*

*HA*, hospital anxiety, *HD* hospital depression, *PCS-12* physical component summary, *MCS-12* mental component summary

^*^Indicates significant result, *P* < 0.001

In addition, both HA and HD were negatively correlated with MCS-12 scores (r = -0.520 and -0.370, respectively, *p* < 0.001), while resilience was positively correlated with MCS-12 scores (r = 0.314, *p* < 0.001). These results suggest that higher anxiety and depression were associated with lower mental health-related QoL, while higher resilience was associated with better mental health-related QoL.

Caregiver burden also showed a negative correlation with PCS-12 and MCS-12 scores (r = -0.377, *p* < 0.001 for both), indicating that higher caregiver burden was associated with lower QoL, both physically and mentally.

### Path analysis

**Fig. 2:**
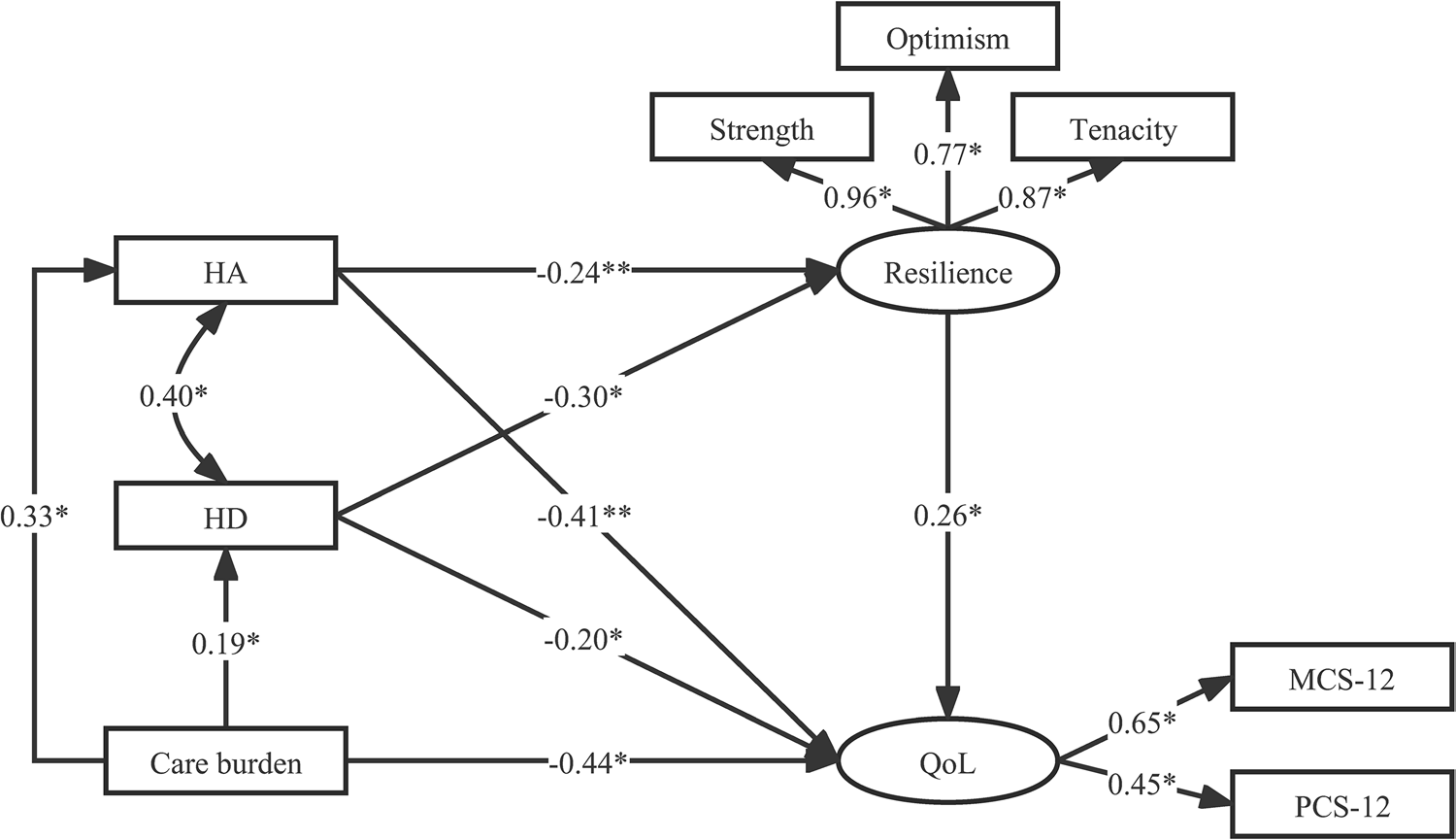
Mediated effects model for QoL. Model adjusted for age, sex, education, Caring duration (years), marital status, family income, only child, chronic disease, and patient support group on social media. *P < 0 .001

Utilizing SPSS Amos, structural equation modeling was employed to evaluate the relationships between care burden, depression, anxiety, individual resilience, and QoL (Fig. 2), whilst controlling for sociodemographic variables. The final extended model demonstrated an excellent fit (X2/df ratio = 3.492, GFI = 0.936, CFI = 0.907, IFI = 0.908, and RMSEA = 0.064). The path "Care burden → resilience" was omitted due to a P-value greater than 0.05.

Care burden significantly impacted QoL both directly and indirectly through its influence on depression, anxiety, and individual resilience, with a mediation effect of 32.21%. Both depression and anxiety affected QoL directly and indirectly through individual resilience, with mediation effects of 27.92% and 13.53%, respectively (Table 3). The four predominant factors influencing QoL, in descending order, were care burden, anxiety, depression, and individual resilience, with effect sizes of -0.652, -0.473, -0.283, and 0.265, respectively (Table 3). Collectively, these variables accounted for 88.00% of the variance in QoL.

**Table 3:** HD, HA, Care burden Resilience of direct, indirect, and total effects on the quality of life

	Total effects (95%CI) ^a^	*P*	Direct effects (95%CI) ^a^	*P*	Indirect effects (95%CI) ^a^	*P*	Mediating effect (%)
HD	-0.283 (-0.382, -0.192)	< 0.001	-0.204 (-0.303, -0.114)	< 0.001	-0.079 (-0.118, -0.051)	< 0.001	27.92%
HA	-0.473 (-0.575, -0.372)	< 0.001	-0.409 (-0.512, -0.308)	< 0.001	-0.064 (-0.095, -0.038)	< 0.001	13.53%
Care burden	-0.652 (-0.752, -0.551)	< 0.001	-0.442 (-0.548, -0.338)	< 0.001	-0.21 (-0.26, -0.168)	< 0.001	32.21%
Resilience	0.265 (0.173, 0.359)	< 0.001	0.265 (0.173, 0.359)	< 0.001	—	—	—

*HD* hospital depression, *HA* hospital anxiety

^a^ Results adjusted for age, sex, education, Caring duration (years), marital status, family income, only child, chronic disease, and patient support group on social media

The results of the structural equation modeling support our hypothesis that care burden, anxiety, and depression significantly affect QoL and that individual resilience mediates these relationships.

## Discussion

The QoL of caregivers is paramount for effective care provision to their loved ones. Caregivers with a good QoL are well-rested, emotionally stable, and supported by social networks, enabling them to deliver high-quality care. Conversely, caregivers with a poor QoL may experience burnout, stress, and exhaustion, impairing their caregiving abilities and resulting in suboptimal care. Our study revealed that adult child caregivers experienced a higher burden, significantly affecting their QoL, primarily through elevated levels of anxiety and depression. These findings align with previous research highlighting the detrimental impact of caregiving on family caregivers' QoL for advanced cancer patients [32, 33]. A body of research has shown that elevated levels of anxiety and depression are associated with a lower quality of life [34]. These psychological conditions often lead to emotional distress, reduced social engagement, and impaired overall well-being. Anxiety and depression can also have significant physical health implications, including increased risk factors for chronic diseases, compromised immune function, and even decreased life expectancy. These factors can further erode a person's QoL [35, 36]. Moreover, our study delved into the mediating role of individual resilience, demonstrating its protective influence on the QoL of cancer caregivers. Healthcare professionals providing care to family caregivers of advanced cancer patients can utilize various strategies to identify the development of resilience in caregivers [37]. Assessing their ability to adapt to and manage caregiving challenges, including problem-solving, emotional regulation, and social support utilization, is crucial. Observing indicators of personal growth and positive adjustment, such as optimism, self-efficacy, and a sense of purpose in their caregiving role, allows professionals to tailor interventions that enhance the QoL of adult child caregivers. Education and guidance on effective coping strategies can be implemented to improve health outcomes for cancer survivors and contribute to the overall well-being of both caregivers and patients [38].

In this study, both depression and anxiety were found to impact QoL directly and indirectly via individual resilience. The prominence of individual resilience in the relationship between caregiver burden and QoL is noteworthy. In the face of substantial care burden, anxiety, and depression, individual resilience can serve as a buffer, safeguarding QoL. This aligns with literature emphasizing resilience as a critical psychological resource for maintaining and improving mental health [39]. Resilience aids in managing the emotional strain, navigating the complexities of caregiving, and effectively mitigating the adverse impacts on QoL. The structural equation modeling results lend credence to our hypothesis, affirming that care burden, anxiety, and depression significantly affect QoL, with individual resilience serving as a critical mediator in these relationships. Given these findings, interventions aiming to enhance individual resilience could prove beneficial in improving caregivers' QoL. This underlines the importance of comprehensive support programs, which not only equip caregivers with essential disease and caregiving knowledge but also foster resilience, thereby mitigating caregivers' burden and improving their QoL. Such resilience-oriented initiatives could go a long way in addressing the psychological and economic strains faced by caregivers.

Our study revealed that only children in families experience increased caregiver burden and overall poor QoL, exacerbated by the physical and emotional demands of caregiving. Being the sole caregiver for an ill parent can be overwhelming for an only child, and the absence of sibling support can lead to feelings of isolation and heightened emotional strain. Recognizing these unique challenges is crucial, and it is essential to provide appropriate support and resources for only child caregivers. With the rise of single-child families worldwide, the mental health concerns of only children facing cancer-related stressors have become more pronounced. Their singular status in the family introduces specific stressors that can be intensified in the context of cancer-related anxiety. Thus, prioritizing the psychological well-being of these individuals and offering tailored support to navigate their specific challenges is vital. Existing research underscores the pervasive psychological distress that affects the nuclear family, impacting the physical and mental well-being of caregivers [8, 40]. Additionally, adult children often shoulder significant filial responsibilities, finding the physical and emotional suffering of their parent and the prospect of bereavement highly stressful [41]. Therefore, a comprehensive understanding and provision of adequate support systems for these individuals are imperative.

Numerous studies underscore the impact of demographic elements such as age, gender, marital status, and social support on caregiver burden [7, 42]. Consistent with prior research, our results indicate that younger caregivers, less equipped to handle caregiving's emotional demands, report lower MCS-12 scores. Our data also reveal lower MCS-12 scores among female and unmarried participants, which could be a reflection of societal norms that often place women at the forefront of caregiving responsibilities, and the amplified stress faced by those lacking spousal support.

Our study demonstrated that patients involved in online health support groups experience heightened anxiety, stress, and more frequent negative emotions. These increased negative emotions can, in turn, detrimentally impact their mental and physical health. Online health support groups, often formed through social media platforms, serve as common grounds for those with similar diagnoses and treatment experiences, facilitating consultation and emotional support [43, 44]. However, these public platforms sometimes inadvertently become outlets for sharing adverse news and emotional distress, potentially contributing to the deterioration of group members' mental health. Further compounding this issue is the occasional lack of professional presence in these groups to provide expert guidance and reassurance, leading to heightened anxiety and distress [39]. Additionally, our findings revealed a correlation between being an only child, participating in virtual patient support groups, and lower MCS-12 scores, indicating poorer mental health outcomes. These results underscore the significance of the caregiving social context, suggesting that virtual support groups may not be as effective as their in-person counterparts in mitigating the mental health burdens associated with caregiving.

We matched the demographic characteristics of the caregivers with those of the cancer patients in Supplemental Table 1. The age distribution between caregivers and cancer patients appears to align with the common age range for cancer diagnosis and caregiving responsibilities. The gender distribution between caregivers and cancer patients is relatively balanced, with slightly more female caregivers. This could be explored further to understand potential gender-related dynamics in caregiving roles. There is a notable difference in the education levels of caregivers and cancer patients. Caregivers appear to have a higher percentage of individuals with bachelor's level education or above.

### Limitation

This study has limitations. Firstly, the use of a convenience sample may limit generalizability to a broader population of adult child caregivers. Secondly, reasons for non-participation of eligible caregivers were not explored, potentially impacting the results and conclusions. Therefore, understanding caregiver refusals and differences between participants and non-participants is important. In addition, the data collected relied on self-reported measures taking place at the time of admission to the hospital for treatment which may introduce response bias, potentially affecting data accuracy.

## Conclusions

This study explored the relationship between caregiver burden and QoL among adult children caring for a parent with advanced cancer. The results indicate that Chinese adult child caregivers experience a burden that directly affects their QoL in a negative manner. However, the study highlights the crucial role of individual resilience as a mediating factor in this relationship. Resilience helps counterbalance the negative impacts of caregiver burden, such as anxiety and depression. These findings underscore the significant impact of caregiving on the physical and mental well-being of adult children supporting cancer patients. To improve the QoL of cancer caregivers, interventions should consider both demographic characteristics and the sociopsychological context in which caregiving takes place.

### Supplementary Information

Below is the link to the electronic supplementary material.

Supplementary file1 (DOCX 18 KB)

## Data Availability

The datasets produced and/or analyzed during this study cannot be made publicly accessible due to ethical restrictions imposed by the First Affiliated Hospital with Nanjing Medical University. However, interested parties may obtain the data from the corresponding author upon reasonable request.
