# Supplementary material for: Enhancing resilient coping strategies for quality of life in Chinese adult children caregiving for parents with advanced cancer: a cross-sectional study
Source: Support Care Cancer. 2023 Sep 26;31(10):591. doi: 10.1007/s00520-023-08057-y (PMC10520155; doi:10.1007/s00520-023-08057-y)
Supplement: Supplementary file 1 — Supplementary file1 (DOCX 18 KB) [file 520_2023_8057_MOESM1_ESM.docx]

Supplemental table 1. Demographic characteristics of the cancer patients who were associated with the participants of the study. (n=614)

| Variables | Number (%) | |
| --- | --- | --- |
| Age (years) |  | |
| <50 | 162 (26.36) | |
| 50~60 | 205 (33.44) | |
| >60 | 247 (40.20) | |
| Sex |  | |
| Male | 320 (52.12) | |
| Female | 294 (47.88) | |
| Education |  | |
| Primary education | 284 (46.25) | |
| Secondary education | 279 (45.44) | |
| Bachelor’s level and above | 51 (8.31) | |
| Marital status |  | |
| Married | 581 (94.68) | |
| Others | 33 (5.32) | |
| Religion |  | |
| No | 532 (86.71) | |
| Yes | 82 (13.29) | |
| Diagnosis | |  |
| Lung cancer | 155 (25.24) | |
| Breast cancer | 98 (15.96) | |
| Gastric cancer | 80 (13.03) | |
| Colon cancer | 62 (10.10) | |
| Liver cancer | 60 (9.77) | |
| Esophageal cancer | 47 (7.65) | |
| Other cancers | 112 (18.24) | |
